# Durvalumab After Chemoradiation for Unresectable Stage III Non-Small Cell Lung Cancer: Inferior Outcomes and Lack of Health Equity in Hispanic Patients Treated With PACIFIC Protocol (LA1-CLICaP)

**DOI:** 10.3389/fonc.2022.904800

**Published:** 2022-07-12

**Authors:** Luis E. Raez, Oscar Arrieta, Diego F. Chamorro, Pamela Denisse Soberanis-Piña, Luis Corrales, Claudio Martín, Mauricio Cuello, Suraj Samtani, Gonzalo Recondo, Luis Mas, Zyanya Lucia Zatarain-Barrón, Alejandro Ruíz-Patiño, Juan Esteban García-Robledo, Camila Ordoñez-Reyes, Elvira Jaller, Franco Dickson, Leonardo Rojas, Christian Rolfo, Rafael Rosell, Andrés F. Cardona

**Affiliations:** ^1^ Thoracic Oncology Program, Memorial Cancer Institute, Florida Atlantic University (FAU), Miami, FL, United States; ^2^ Thoracic Oncology Unit and Personalized Oncology Laboratory, National Cancer Institute (INCan), Mexico City, Mexico; ^3^ Foundation for Clinical and Applied Cancer Research (FICMAC), Bogotá, Colombia; ^4^ Molecular Oncology and Biology Systems Research Group (Fox-G), Universidad el Bosque, Bogotá, Colombia; ^5^ Thoracic Oncology Unit, Centro de Investigación y Manejo del Cáncer – CIMCA, San José, Costa Rica; ^6^ Thoracic Oncology Unit, Alexander Fleming Institute, Buenos Aires, Argentina; ^7^ Medical Oncology Department, Hospital de Clínicas, Universidad de la Republica – UdeLAR, Montevideo, Uruguay; ^8^ Medical Oncology Department, Clinica Las Condes, Santiago, Chile; ^9^ Thoracic Oncology Unit, Centro de Educación Médica e Investigaciones Clinicas (CEMIC), Buenos Aires, Argentina; ^10^ Medical Oncology Department, Instituto Nacional de Enfermedades Neoplásicas – INEN, Lima, Peru; ^11^ Division of Hematology/Oncology, Mayo Clinic, Scottsdale, AZ, United States; ^12^ Clinical Oncology Department, Clínica Colsanitas, Bogotá, Colombia; ^13^ Thoracic Oncology Center, Tisch Cáncer Center, Mount Sinai Hospital System & Icahn School of Medicine, Mount Sinai, New York, NY, United States; ^14^ Cancer Biology and Precision Medicine Program, Germans Trias i Pujol Research Institute (IGTP)/Dr. Rosell Oncology Institute (IOR) Quirón-Dexeus University Institute, Barcelona, Spain; ^15^ Direction of Research, Science and Education, Luis Carlos Sarmiento Angulo Cancer Treatment and Research Center (CTIC), Bogotá, Colombia

**Keywords:** durvalumab, non-small cell lung cancer, hispanics, survival, health equity, immunotherapy

## Abstract

**Objectives:**

To compare the rate disparity between outcomes (overall survival (OS), progression-free survival (PFS), and safety) of concurrent chemoradiation (cCRT) followed by durvalumab in two patient cohorts with locally advanced (LA) stage III non-small cell lung cancer (NSCLC), one non-Hispanic White (NHW), and the other Latin-American.

**Methods:**

A multicenter retrospective study was performed, including 80 Hispanic and 45 NHW LA stage III NSCLC patients treated with cCRT followed by durvalumab. Both cohorts were analyzed in terms of main outcomes (OS, PFS, and safety) and compared between them and with the PACIFIC trial population outcomes. The efficacy-effectiveness gap was assessed using an efficacy-effectiveness (EE) factor that was calculated by dividing each cohort median overall survival by the corresponding reference OS from the PACIFIC trial. In both cohorts, results of PD-L1 testing were recorded, and the main outcomes were compared according to PD-1 expression levels (≥50%, 1–49%, and <1%).

**Results:**

For the entire population (N=125), the overall response rate (ORR) was 57.6% (N=72), and 18.4% (N=25) achieved stable disease. OS was 26.3 months (95%CI 23.9-28.6), and PFS was 20.5 months (95%CI 18.0-23.0). PFS assessed by ethnicity showed a median for the Hispanic population of 19.4 months (95%CI 16.4-22.5) and 21.2 months (95%CI 17.2-23.3; p=0.76) for the NHW group. OS by race showed a significant difference in favor of the NHW group, with a median OS of 27.7 months (95%CI 24.6-30.9) vs. 20.0 months (95%CI 16.4-23.5) for Hispanics. (P=0.032). Unadjusted 12-month and 24-month OS was 86.6% (95%CI 79.9–88.0) and 46.6% (95%CI 40.2–48.3) for NHW compared to 82.5% (95%CI 77.1–84.2) and 17.5% (95%CI 15.6-24.5) in Hispanics. NHW had an EE factor of 0.78 and Hispanics had 0.58, showing a reduction in survival versus NHW and PACIFIC of 20% and 42%, respectively. HR for the OS among NHWs and Hispanics was 1.53 (95%CI 1.12-1.71; P=0.052) and 2.31 (95%CI 1.76-2.49; P=0.004). Fifty-six patients (44.8%) had some degree of pneumonitis due to cCRT plus durvalumab. There was no difference in the proportion of pneumonitis according to race (P=0.95), and the severity of pneumonitis was not significantly different between Hispanics and NHWs (P=0.41).

**Conclusions:**

Among patients with LA stage III NSCLC, NHW had better survival outcomes when compared to Hispanics, with an OS that seems to favor the NHW population and with an EE factor that shows a shorter survival in Hispanics compared with NHW and with the PACIFIC trial group.

## Highlights

• Chemoradiation followed by durvalumab is the standard of care in locally advanced stage III NSCLC.
• Outcomes of this treatment are not evaluated in Hispanic patients and could be inferior compared with non-Hispanic whites and even more with the results shown in the registry trial (PACIFIC).
• Reasons for inferior results in Hispanic patients must be evaluated and analyzed in prospective trials and could be related to delays in starting durvalumab after chemoradiation treatment.

## Introduction

Lung cancer (LC) has been one of the leading causes of cancer-related deaths during the last years in the United States, and it continues to be one of the leading causes of cancer-related deaths in men and women worldwide (1). Among LC, non-small cell lung cancer (NSCLC) accounts for 85% of all cases, with 1.28 million diagnoses made between 2007 and 2017 (2, 3). In the US, nearly 30% of patients with NSCLC are diagnosed with locally advanced disease (Stage III). This stage represents a complex group of patients with diverse characteristics regarding the extension of the disease, prognosis, and possible management that goes from resectable to unresectable lesions (4, 5). In patients with unresectable disease, platinum-based chemotherapy with concurrent chemotherapy has been the standard of care (6).

However, in 2017, the PACIFIC trial changed the treatment paradigm for locally advanced NSCLC. This study demonstrated a significant improvement in progression-free survival (PFS) and overall survival (OS) among patients who received durvalumab (anti-PD-L1) in addition to concurrent chemoradiotherapy (cCRT) (7). The updated 5-year analysis of the PACIFIC trial remained consistent with the current outcomes and showed a PFS of 33.1% and an OS of 42.9% in the durvalumab arm (8). In addition, durvalumab has been demonstrated to be safe, with pneumonitis as the main adverse effect (4.4%) (9).

Despite the clear evidence of benefits with immunotherapy in locally advanced NSCLC, most clinical trials have been done in Non-Hispanic Whites (NHW), leaving aside other population groups such as Hispanics. There are many disparities in the outcomes of Hispanic patients compared with NHW when they are treated with immunotherapy. These disparities begin with differential access to optimal cancer care and treatment, molecular profiling, or follow-up (10, 11).

To provide some insights into the disparities between Hispanics and NHW in the outcomes of NSCLC treatment, we designed a multicenter retrospective study that included both populations and compared the outcomes after treatment with durvalumab in addition to cCRT.

## Methods

### Study Design and Patients

This multicenter retrospective study included 80 Hispanic patients with histologically and/or cytologically confirmed unresectable stage III NSCLC who received at least one cycle of consolidative durvalumab post-cCRT, after reaching stable disease. All were treated in fourth-level centers in Florida (United States), Mexico, Central America, and Colombia between February 2018 and December 2021. To compare the rate of disparity in outcomes, the results of Hispanic patients were compared to a cohort of non-Hispanic white (NHW) patients (N=45) treated in the United States (at Memorial Cancer Institute, part of Memorial Healthcare System, Miami, FL), assuming that their results were homogeneous with those presented in the PACIFIC study (12) (**Supplementary Figure S1**). An independent review board approved the study in Bogotá Colombia (Kayre/FICMAC IRB 2018-14-021), and institutional approval of each linked site was subsequently obtained. In addition, the study was conducted in accordance with the Declaration of Helsinki. In each case, cCRT was administered with curative intent (54-66 Gy) concurrently with platinum-based chemotherapy for at least two cycles, followed by immunotherapy with durvalumab for one year (10 mg/kg intravenously every 2 weeks). Radiotherapy administered to patients was homogeneous, and all patients were treated with radiation in reference centers of main cities in Latin America using intensity-modulated radiation therapy (IMRT) (13). Furthermore, all participating radiotherapy centers have radiation protocols under ASTRO/ESTRO recommendations (14). Treatment patients with EGFR mutations (N=6) or ALK translocations (N=1) were allowed to be included, and each treating physician chose the concurrent chemotherapeutic regimen. The simulation procedure for RT planning and the definitions of target volumes followed previous recommendations and descriptions (15, 16). Follow-up chest CT was performed 1 month after cCRT, positron emission tomography (PET)-CT was done at diagnosis (92% of cases) and 3-4 months after the completion of cCRT (when available), and chest CT was repeated every 3 months after completion of CCRT as follow-up.

Globally, information was collected on tumor status, age, sex, Eastern Cooperative Oncology Group (ECOG) performance status, smoking history, number of pack-year, baseline lung comorbidities (asthma, chronic obstructive pulmonary disease, interstitial lung disease), histology subtype, cancer stage, PD-L1 expression status, platinum type, and time from cCRT completion to ICI start date. Information regarding the main adverse effects, with particular emphasis on post-cCRT pneumonitis, was also collected. The study estimated progression-free survival (PFS), overall response rate (ORR), overall survival (OS), and the primary outcomes obtained with the second line. The analysis of the results was stratified according to the expression of PD-L1 and according to RECIST-1.1 (17).

The PFS was defined as the last date of cCRT until radiographically confirmed progression or death. OS was defined as the time from treatment to death or loss to follow-up. Radiation pneumonitis was diagnosed clinically based on the presence of classic symptoms, timing, history of radiation therapy, imaging findings, and exclusion of alternative causes, such as infection, cardiogenic edema, pulmonary embolism, drug-induced pneumonitis, and other causes. Radiation pneumonitis may be graded using the Common Toxicity Criteria for Adverse Events (Version 5.0) (Common Terminology Criteria for Adverse Events (CTCAE) |, Protocol Development |, CTEP (2000). Retrieved from, https://ctep.cancer.gov/protocolDevelopment/electronic_applications/ctc.htm.) (18).

### PD-L1 Testing

PD-L1 expression was determined by immunohistochemistry using the Dako 22C3 pharmDx kit, with more than 100 tumor cells present in the slide section for accurate PD-L1 readings. PD-L1 testing was completed on biopsies taken at diagnoses. Patients were grouped according to PD-L1 status (i.e., ≥50%, 1–49%, and <1% subgroups) for survival analyses. Patients with unknown PD-L1 expression status were also included in this study to reflect real-world durvalumab use.

### Statistical Analysis

All analyses were conducted on IBM SPSS Statistics software version 25.0 (SPSS Inc. Chicago, IL, USA). Descriptive analyses were utilized to provide an overview of the characteristics of the study population. Categorical variables were assessed *via* the Chi-Square test or, whenever appropriate, Fisher’s Exact test. OS and PFS were reported as Kaplan Meier survival curves. Multivariable Cox regression models were generated to assess potential confounders. Two-sided P-Value was set to determine statistically significant outcomes. There were no adjustments made for multiple comparisons, and in all cases, the significance level was P=0.05. The efficacy-effectiveness gap was assessed using an efficacy-effectiveness (EE) factor that was calculated by dividing each cohort’s median overall survival by the corresponding reference OS from the most recent report from PACIFIC (12). This factor was used to estimate the presence of an EE gap and compare the real-world population’s survival relative to the clinical trial population. An EE factor of 0.60 indicates that median survival is 40% shorter in clinical practice than in the reference clinical trial (19).

## Results

### Patients, Tumors, and Treatment Characteristics

Eighty Hispanic patients and 45 NHW were included. Baseline patient and treatment characteristics are summarized in **Table 1**. To establish the comparability of clinical variables between Hispanic patients treated in the US and Latin American countries, a stratified analysis was performed for age (P=0.53), gender (P=0.71), baseline performance status (P=0.22), and place of origin (P=0.57) without finding statistically significant differences. This allowed consideration of a balanced intervention for both populations to carry out a correct analysis of the disparities between groups. At the time of diagnosis, 52% of the patients were ≥65 years old, the majority were women (52.8%), and the most frequent histology was adenocarcinoma (80%). Two-thirds of the non-smoking patients were women (14/21), and six of them had EGFR mutations (four with exon 19 deletions and two with the L858R mutation). Overall, 19 (15.2%), 35 (28.0%), and 65 (52.0%) patients had PD-L1 expression ≥50%, <1%, and between 1%-49%, respectively. Most patients had a good ECOG performance status; 60% received carboplatin as part of the cCRT regimen, especially in combination with paclitaxel (40%) or pemetrexed (20%). The mean time from cCRT completion to durvalumab initiation was 37.3 days (SD ±23.7, range 7-133 days). There were no statistically significant imbalances between PD-L1 subgroups. Still, a significant difference was found in Hispanics regarding using the cisplatin/pemetrexed combination, while NHW were more exposed to cisplatin/etoposide (P=0.002). Similarly, the mean time interval between cCRT and the start of durvalumab was significantly shorter in NHW (difference of 10 days less, P=0.02). The median follow-up for the entire cohort of included patients was 19.6 months (95%CI 8.1-39.2).

**Table 1:** Baseline patient and treatment characteristics.

Variable	All N = 125 (%)	Hispanic N = 80 (%)	Non-Hispanic whites N = 45 (%)	P-value
**Age**				
Median ≥65 years <65 years	66 (41-90) 65 (52.0) 60 (48.0)	64 (41-90) 39 (48.8) 41 (51.2)	66 (46-90) 26 (57.8) 19 (42.2)	0.35
**Gender**				
Male Female	59 (47.2) 66 (52.8)	38 (47.5) 42 (52.5)	21 (46.7) 24 (53.3)	0.92
**Histology**				
Adenocarcinoma SCC	100 (80.0) 25 (20.0)	62 (77.5) 18 (22.5)	38 (84.8) 7 (15.6)	0.48
**ECOG**				
0 1	86 (68.8) 39 (31.2)	51 (63.7) 29 (36.3)	35 (77.8) 10 (22.2)	0.11
**Smoking history**				
Current Former Never	9 (7.2) 95 (76.0) 21 (16.8)	6 (7.5) 59 (73.8) 15 (18.8)	3 (6.7) 36 (80.0) 6 (13.3)	0.71
**Stage**				
IIIA IIIB IIIC	35 (28.0) 70 (56.0) 20 (16.0)	20 (25.0) 44 (55.1) 16 (20.0)	15 (33.3) 26 (57.8) 4 (8.9)	0.31
**Baseline lung** **Comorbidities**				
COPD ILD	15 (12.0) 3 (2.4)	12 (15.0) 2 (2.5)	3 (6.7) 1 (2.2)	0.38
**Other comorbidities**				
Yes No	43 (34.4) 82 (65.6)	34 (42.5) 46 (57.5)	20 (44.4) 25 (66.6)	0.35
**PD-L1 expression**				
≥50% 1-49% <1% ND	19 (15.2) 65 (52.0) 35 (28.0) 6 (4.8)	15 (18.8) 38 (47.5) 21 (26.3) 6 (7.5)	4 (8.9) 27 (60.0) 14 (31.1)	0.097
**EGFR mutation**				
Yes No ND	6 (4.8) 117 (94.4) 2 (1.6)	4 (5.0) 76 (95.0)	2 (4.4) 41 (91.2) 2 (4.4)	0.43
**Platinum Type**				
Carboplatin Cisplatin	75 (60.0) 50 (40.0)	53 (66.3) 27 (33.7)	22 (48.9) 23 (51.1)	0.072
**Chemotherapy combination**				
Carboplatin/Paclitaxel Carboplatin/Pemetrexed Cisplatin/Pemetrexed Cisplatin/Etoposide	50 (40.0) 25 (20.0) 43 (34.4) 7 (5.6)	39 (48.8) 14 (17.5) 21 (26.2) 6 (7.5)	11 (24.4) 11 (24.4) 1 (2.2) 22 (48.9)	0.002
**Type of chemoradiotherapy**				
Sequential Concurrent	8 (6.4) 117 (93.6)	6 (7.5) 74 (92.5)	2 (4.4) 43 (95.6)	0.71
**Time from CRT completion to durvalumab** (mean ± SD in days)	37.3 ( ± 23.7)	39.0 ( ± 17.3)	29.1 ( ± 11.1)	0.02
**The compliance rate with radiotherapy (%)**	90.0	86.0	95.0	0.32

SCC, squamous cell carcinoma; ECOG, Eastern Cooperative Oncology Group; COPD, chronic obstructive pulmonary disease; ILD, interstitial lung disease; ND, no data.

### Survival Outcomes

**Figure 1:**
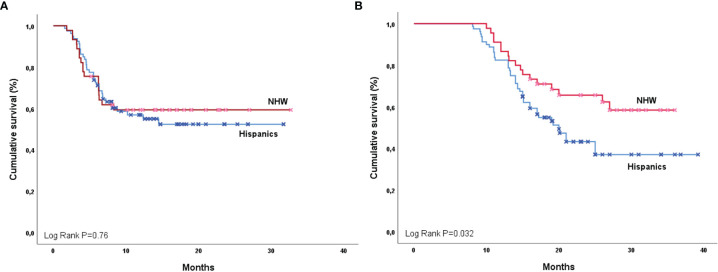
Progression-free survival **(A)** and overall survival **(B)** by ethnicity (Hispanic and NHW).

For the 125 patients, the overall response rate (ORR) was 57.6% (N=72), and 18.4% (N=25) achieved stable disease (SD). OS was 26.3 months (95%CI 23.9-28.6) (**Supplementary Figure S2A**), and PFS was 20.5 months (95%CI 18.0-23.0) (**Supplementary Figure S2B**). When PFS was assessed by ethnicity, the median for the Hispanic population was 19.4 months (95%CI 16.4-22.5) and 21.2 months (95%CI 17.2-23.3; P=0.76) for the NHW group (**Figure 1A**). However, analysis of OS showed a significant difference in favor of the NHW group, given the median was 27.7 months (95%CI 24.6-30.9) versus 20.0 months (95%CI 16.4-23.5) for Hispanics. (P=0.032) (**Figure 1B**). Unadjusted 12-month and 24-month OS was 86.6% (95%CI 79.9–88.0) and 46.6% (95%CI 40.2–48.3) for NHW compared to 82.5% (95%CI 77.1–84.2) and 17.5% (95%CI 15.6-24.5) in Hispanics.

Among Hispanics, PFS was higher in those with better ECOG [ECOG 0: 21.4 months (95%CI 17.8-25.1) vs. ECOG 1: 10.2 months (95%CI 4.6-15.7); P=0.19] (**Supplementary Figure S3**), in patients with SCC [25.5 months (95%CI 20.2-30.8) vs. Adenocarcinomas 15.5 (CI95% 12.8-18.3); P=0.06] (**Supplementary Figure S4**) and in those with higher PD-L1 expression [PD-L1 ≥50% PFS NR, PD-L1 1-49% 14.5 months (95%CI 8.8-NR) and PD-L1 <1% 12.3 months (95%CI 6.8-13.6); P=0.001]. Neither history of tobacco exposure (P=0.67), tumor stage (P=0.10), nor presence of pneumonitis (P=0.51) influenced PFS among Hispanics. For the NHW group, the only variable that influenced PFS was the level of PDL-1 expression [PD-L1 ≥50% PFS NR, PD-L1 1%-49% 13.3 months (95%CI 11.9-NR) and PD-L1 <1% 10.4 months (95%CI 9.8-14.6); P=0.018)].

**Figure 2:**
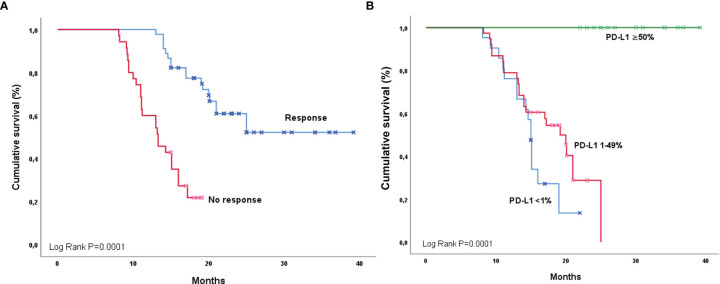
Overall survival among Hispanics according to response **(A)** and PD-L1 expression **(B)**.

**Figure 3:**
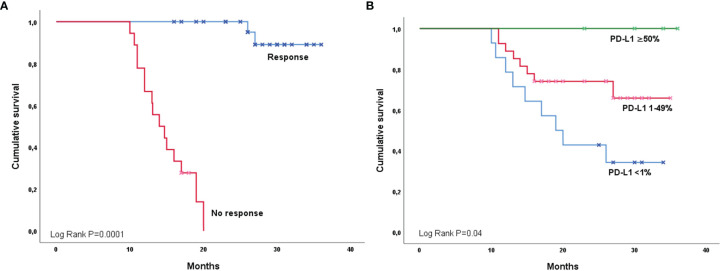
Overall survival among NHWs according to response **(A)** and PD-L1 expression **(B)**.

Univariate analysis for OS revealed that overall response to CRT positively impacted the survival in both Hispanics [OS responders 29.2 months (95%CI 25.8-37.7) vs. Non-responders 13.3 months (95%CI 10.3-16.2); P=0.0001] (**Figure 2A**) and NHW groups [OS responders 34.9 months (95%CI 33.6-36.3) vs. Non-responders 14.8 months (95%CI 13.1-16.4); P=0.0001] (**Figure 3A**). Similarly, Hispanic patients [PD-L1 ≥50% OS NR, PD-L1 1%-49% 25.0 months (95%CI 19.2-NR), and PD-L1 <1% 19.0 months (95%CI 13.0-16.1); P=0.0001] (**Figure 2B**) and NHW [PD-L1 ≥50% OS NR, PD-L1 1-49% 24.0 months (95%CI 14.7-NR) and PD-L1 <1% 19.0 months (95%CI 13.0- 16.8); P=0.04] (**Figure 3B**) with higher PD-L1 expression had better OS. Neither ECOG, smoking history, tumor staging, histology, nor pneumonitis influenced OS in either group. In the multivariate model for OS, the only predictor of increased mortality was lack of response after CRT (HR 7.8, 95%CI 3.1-19.4). In contrast, the only factor that positively impacted OS among Hispanics and NHW was PD-L1 expression ≥50% compared to the PD-L1 <1% group (HR 0.69, 95%CI 0.50-0.97). 08). In the model for PFS, the only predictor for a better outcome among Hispanics and NHW was PD-L1 expression ≥50% (HR 0.55, 95%CI 0.37-0.81). The OS (P=0.52) and PFS (P=0.40) of patients carrying EGFR mutations did not differ significantly from the *Wt* population.

To compare data derived from the PACIFIC study with real-life Hispanics and NHW treated with CRT and durvalumab in our research, the efficacy-effectiveness factor and hazard ratio for OS (between 24 and 36 months of follow-up) was estimated, comparing both groups to the durvalumab arm in the PACIFIC. NHW had an EE factor of 0.78, indicating that median OS was 22% shorter for those patients treated in clinical practice than median OS from the registered clinical trial receiving the same treatment. In addition, the EE factor for Hispanics was 0.58, showing a reduction in survival versus NHW and PACIFIC of 20% and 42%, respectively. The corresponding HR for the OS among NHW and Hispanics was 1.53 (95%CI 1.12-1.71; P=0.052) and 2.31 (95%CI 1.76-2.49; P=0.004), respectively.

### Safety Analysis

In the general population, 56 patients (44.8%) had some degree of pneumonitis due to CRT plus durvalumab. Pneumonitis was grade 1, 2, and 3 in 51.8% (N=29), 35.7% (N=20), and 12.5% (N=7), respectively. There was no difference in the proportion of pneumonitis according to race (P=0.95), previous tobacco exposure (P=0.14), type of chemotherapy regimen (P=0.36), or history of pulmonary comorbidity (P=0.55). Similarly, the severity of pneumonitis was not significantly different between Hispanics and NHW (P=0.41) and was not response-dependent (P=0.24).

## Discussion

It is essential to do real-world studies with diverse ethnic populations to address cancer disparities, reduce the variability of the results among minorities, and promote global access oncology. In the original PACIFIC trial, a landmark study that changed the standard of care in Stage IIII NSCLC, less than 2% of enrolled patients were documented as a minority, and there was no information about Hispanic ethnicity. In our multicenter retrospective study, we reported the outcomes of cCRT in addition to durvalumab for stage III NSCLC in two populations, Hispanics and NHW. Some studies have shown that the Hispanic population might have worse immunotherapy outcomes, possibly due to a complex interaction of factors such as culture, genomic heritage, or social determinants of health. Compared with NHW, the median PFS among Hispanics was lower but not significant (P=0.76). Nevertheless, when we analyzed OS stratified by race, we found that NHW reached a higher OS than Hispanics. This finding contrasts with previous studies that found no statistical difference in OS between the two ethnic groups (20).

Interestingly, the PFS and OS were higher in the subgroups with increased expression of PD-L1 in both the Hispanic and NHW groups. This result is consistent with the observations done by Kartolo et al., who found that high expression of PD-L1 was associated with improved survival as an independent prognostic factor (21). However, the most evident benefit was observed in a patient with PD-L1 expression of >50%, with no impact in the groups of PD-L1 expression of 1-49% or <1% (21) and, for our study, the median was not reached for the PDL-1 >50% subgroup. Regardless of the expression, there is evidence that durvalumab has a positive impact on outcomes even with PD-L1 expression higher than 1 to 25% (22, 23). Some evidence suggests that among Hispanics, expression in stage IIIB/IV NSCLC is around 21.7% (24).

Our study reports that overall response to cCRT positively impacts the OS, and the benefit is higher for NHW than for Hispanics (34.9 months vs. 29.2 months, respectively). Previously, some studies documented that complete or partial response to the treatment relates directly to an increased OS (25, 26). In this scenario, the outcome is still better for NHW despite the grade of response.

The results demonstrate that the use of durvalumab consolidation among both Hispanics and NHW is associated with improvement in the OS. When we contrasted the results of our study with those of the PACIFIC trial, we found that among NHW, the OS was slightly inferior in clinical practice (EE gap 0.78). Still, for Hispanics, the median survival was significantly shorter than for NHW (20%) and with the PACIFIC intervention (42%). In real-life scenarios, it has been described that the OS tends to be lower (27). Besides the differences between Hispanics and NHW, the inferior survival in both groups could be attributed to a delayed durvalumab onset and a significantly shorter time in favor of NHW. *Post hoc* analysis of the PACIFIC trial suggests that starting the ICI within 14 days after cCRT is associated with a higher OS (28). Also, the follow-up of the patients was relatively short.

In the general population, 56 patients (44.8%) had some degree of pneumonitis due to cCRT plus durvalumab. Pneumonitis was grade 1, 2, and 3 in 51.8% (N=29), 35.7% (N=20), and 12.5% (N=7), respectively. There was no difference in the proportion of pneumonitis according to race (P=0.95), previous tobacco exposure (P=0.14), type of chemotherapy regimen (P=0.36), or history of pulmonary comorbidity (P=0.55). Similarly, the severity of pneumonitis was not significantly different between Hispanics and NHW (P=0.41) and was not response-dependent (P=0.24).

In terms of safety, pneumonitis represents the most severe and life-threatening adverse effect related to immunotherapy (26). We reported a higher pneumonitis incidence than the PACIFIC trial (44.8% in the general population) (7). However, most cases were mild to moderate, without any patients needing to stop immunotherapy. This study did not find any variables related to a higher incidence of pneumonitis among the subjects. Some studies failed to identify specific risk factors associated with the development of pneumonitis among patients treated with durvalumab (29).

As we exposed earlier, PFS did not differ between the populations; however, the OS did. This finding could be explained in light of multiple differences between Hispanics vs. NHW, including overall access to second-line therapy or follow-up. Unfortunately, our available data related to the treatment approach after initial therapy is scarce and unbalanced between the two groups in our cohort. Further analyses are required to find a possible impact of these new variables in the response to therapy. In addition, we would like to remark that due to the immortality bias, commonly present in lung cancer scenarios, it is frequent that many patients exposed to ICI have a measurable effect on the OS but not in the PFS (30). On the other hand, populations with EGFR mutations (among others) should be analyzed independently (31).

Limitations in our analysis include a relatively short follow-up period for patients; however, in the same period, we were able to distinguish the differences between the Hispanic and NHW groups compared to the PACIFIC trial results. Furthermore, we only considered patients treated with cCRT plus durvalumab, which could create a selection bias in the study because we did not compare our results with a control population. In addition, the analysis of basal characteristics of the Hispanic patients treated in Latin America and those treated in the US did not show any differences, and this could be a risk for the interpretation of the data. On the other hand, the specific dose of durvalumab was not actively recorded, and information about other immuno-mediated side effects besides pneumonitis was not homogeneous.

## Conclusion

Among patients with stage III NSCLC, NHW have better survival outcomes when compared to Hispanics. With an OS that seems to favor the NHW population and an EE factor that shows a shorter survival in Hispanics in comparison with NHW and with the PACIFIC trial group. Further analyses must be done to identify factors that might lead to these differences between Hispanics and NHW, and large clinical trials must include more representation of Hispanics.

## Data Availability Statement

The datasets presented in this article are not readily available because of the Colombian organic law of data protection that limits access to raw genetic information in an open format. Requests to access the datasets should be directed to the corresponding author, who will release it upon formal request to the Ministry of Health of Colombia following the requirements of Law 1581 of 2012, paragraph 201811601170851 of 2018.

## Ethics Statement

The studies involving human participants were reviewed and approved by Kayre/FICMAC IRB 2018-14-021. The patients/participants provided their written informed consent to participate in this study.

## Author Contributions

Conception and study design: LER, OA, CR, RR, AC, LC, CM, and LR. Data acquisition: LR, AC, DC, FD, JG-R, EJ, CO, and AR-P. Analysis and Data interpretation: AR-P, AC, LR, OA, DC, CM, MC, SS, GR, LZ-B, and LM. Article Draft: AFC, DFC, LR, OA, LC, CM, MC, SS, GR, LM, LZB, CR, and RR. Manuscript preparation and approval: All authors.

## Funding

Supported by The CLICaP and The Foundation for Clinical and Applied Cancer Research- FICMAC (Bogotá, Colombia) research grant 037-2021.

## Conflict of Interest

LR discloses research support from Merck Sharp & Dohme, Boehringer Ingelheim, Roche, Bristol-Myers Squibb, Pfizer, Novartis, LOXO Pharmaceuticals, Guardant Health, Natera, Syndax, Eli Lilly, and Astra Zeneca. AC discloses financial research support from Merck Sharp & Dohme, Boehringer Ingelheim, Roche, Bristol-Myers Squibb, and The Foundation for Clinical and Applied Cancer Research – FICMAC. Additionally, he was linked and received honoraria as an advisor, participated in speakers’ bureau, and gave expert testimony to Merck Sharp & Dohme, Boehringer Ingelheim, Roche, Bristol-Myers Squibb, Pfizer, Novartis, Celldex Therapeutics, Foundation Medicine, Eli Lilly, and Foundation for Clinical and Applied Cancer Research – FICMAC. OA reports personal fees from Pfizer, grants and individual fees from Astra Zeneca, grants and individual fees from Boehringer-Ingelheim, personal fees from Lilly, individual fees from Merck, and personal fees from Bristol Myers Squibb, grants and personal fees from Roche, outside the submitted work.

The remaining authors declare that the research was conducted in the absence of any commercial or financial relationships that could be construed as a potential conflict of interest.

## Publisher’s Note

All claims expressed in this article are solely those of the authors and do not necessarily represent those of their affiliated organizations, or those of the publisher, the editors and the reviewers. Any product that may be evaluated in this article, or claim that may be made by its manufacturer, is not guaranteed or endorsed by the publisher.
