# Supplementary figures and images for: Durvalumab After Chemoradiation for Unresectable Stage III Non-Small Cell Lung Cancer: Inferior Outcomes and Lack of Health Equity in Hispanic Patients Treated With PACIFIC Protocol (LA1-CLICaP)
Source: Front Oncol. 2022 Jul 12;12:904800. doi: 10.3389/fonc.2022.904800 (PMC9321635; doi:10.3389/fonc.2022.904800)

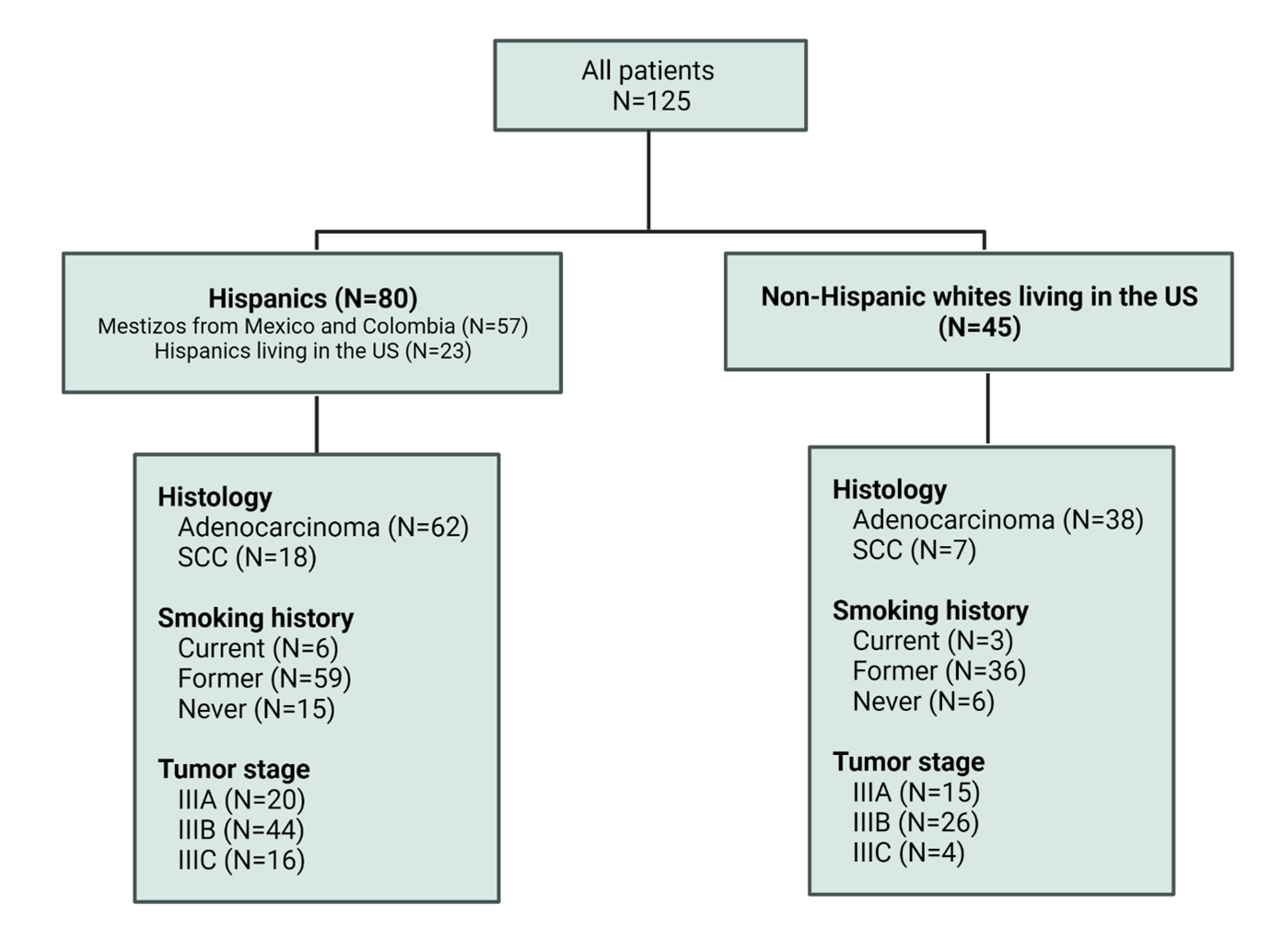

Supplement: Supplementary file 1 [file Image_1.png]

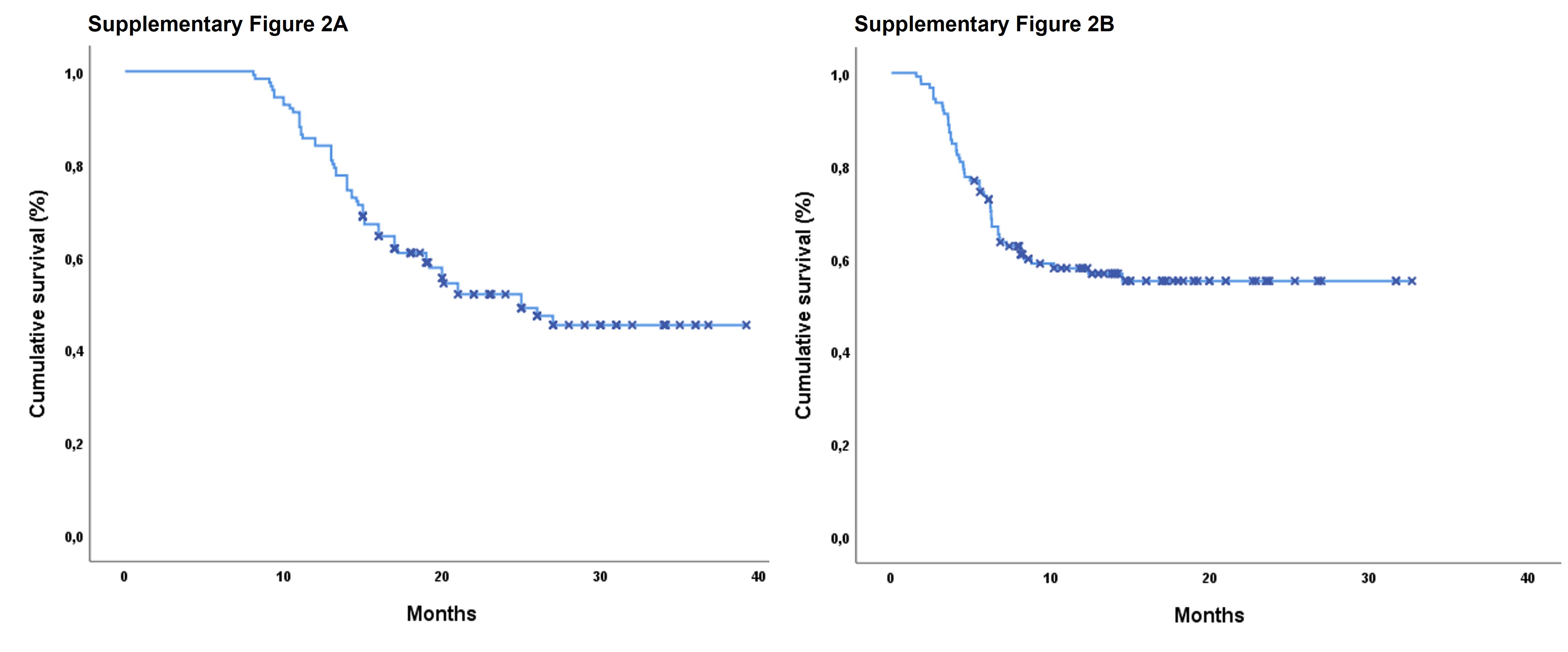

Supplement: Supplementary file 2 [file Image_2.jpeg]

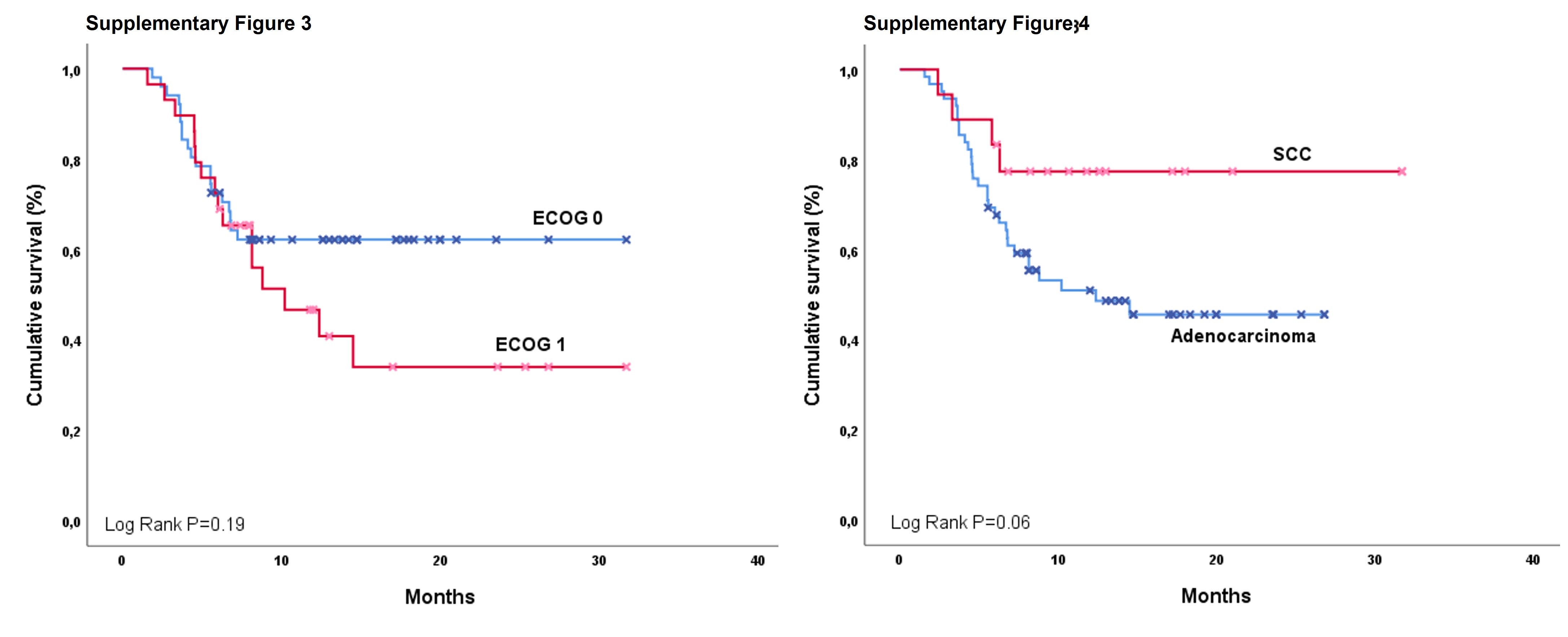

Supplement: Supplementary file 3 [file Image_3.jpeg]
